# The National Pension Fund

**Published:** 1887-08-27

**Authors:** 


### The National Pension Fund.

We publish below a Second List of Institutions from which names have been received by Mr. Howard J. Collins, in support of the formation of a "National Pension Fund for Hospital Officials and Trained Nurses." The figures in parentheses denote the number of names received during July, 1887.

St. Mary's Hospital (56).
Seamen s Hospital, Greenwich (1).
Monsall Hospital, Manchester (37).
Bradford Hospital (2).
Macclesfield Infirmary (21).
Royal Infirmary, Bristol (4).
St. Mary Abbott's Infirmary, Kensington (1).
North-Eastern Hospital for Children (14).
Frome Cottage Hospital (1).
St. Helen's Cottage Hospital (1).
Kensington and Chelsea School, Epsom (2).
Dr. Barnardo's Home, Bow Road (1).
Staffordshire Institution of Nurses (51).
London Association of Nurses, Bond Street (9).
St. Thomas's Home (6).

Also seven from private nurses and others, who do not mention the name of any institution with which they may be connected.

